# Visual and Tactile Sensory Systems Share Common Features in Object Recognition

**DOI:** 10.1523/ENEURO.0101-21.2021

**Published:** 2021-10-04

**Authors:** Sepideh Tabrik, Mehdi Behroozi, Lara Schlaffke, Stefanie Heba, Melanie Lenz, Silke Lissek, Onur Güntürkün, Hubert R. Dinse, Martin Tegenthoff

**Affiliations:** 1 Department of Neurology, BG-University Hospital Bergmannsheil, Ruhr University Bochum, 44789 Bochum, Germany; 2 Institute of Cognitive Neuroscience, Department of Biopsychology, Faculty of Psychology, Ruhr University Bochum, 44780 Bochum, Germany; 3 Neural Plasticity Laboratory, Institute for Neuroinformatics, Ruhr University Bochum, 44780 Bochum, Germany

**Keywords:** shape features, shape perception, similarity judgment, virtual reality, visual and tactile perception

## Abstract

Although we use our visual and tactile sensory systems interchangeably for object recognition on a daily basis, little is known about the mechanism underlying this ability. This study examined how 3D shape features of objects form two congruent and interchangeable visual and tactile perceptual spaces in healthy male and female participants. Since active exploration plays an important role in shape processing, a virtual reality environment was used to visually explore 3D objects called digital embryos without using the tactile sense. In addition, during the tactile procedure, blindfolded participants actively palpated a 3D-printed version of the same objects with both hands. We first demonstrated that the visual and tactile perceptual spaces were highly similar. We then extracted a series of 3D shape features to investigate how visual and tactile exploration can lead to the correct identification of the relationships between objects. The results indicate that both modalities share the same shape features to form highly similar veridical spaces. This finding suggests that visual and tactile systems might apply similar cognitive processes to sensory inputs that enable humans to rely merely on one modality in the absence of another to recognize surrounding objects.

## Significance Statement

Human brains are able to precisely and rapidly identify tactile and visual objects, an ability indicating that we use visual and tactile information interchangeably to recognize surrounding objects. This study examined the role of shape features that enable human reliance on visual or tactile sensory modalities for object recognition and provides evidence that the visual and tactile modalities not only generate two highly congruent perceptual spaces but also use the same shape features to recognize a novel object. This finding contributes to explaining why visual and tactile senses are interchangeable.

## Introduction

Our ability to correctly and quickly recognize an object in both the tactile and visual modalities raises the question of how humans form representations of their surroundings using the visual or tactile system, as well as which common object features play a role in object perceptions to mediate that interchangeability?

Shape is a crucial feature for efficiently interacting with objects in both the visual and tactile domains (Rosch, 1988). While much is known about visual shape processing (Haushofer et al., 2008; Peelen et al., 2014), less information is available regarding tactile shape processing (Klatzky et al., 1985; Hernández-Pérez et al., 2017; Metzger et al., 2019). A series of studies comparing visual and tactile perceptual spaces with familiar objects have revealed that the human perception of familiar objects is not solely determined by the physical features of objects but is influenced by high-level cognitive abilities, including memory (Amedi et al., 2002; Norman et al., 2008; Haag, 2011; Metzger and Drewing, 2019) and prior knowledge of objects for integrating sensory systems (Ernst and Bülthoff, 2004). While other studies have used parametric shape models, such as shell-shaped 3D objects (Gaißert et al., 2008, 2010a, 2011; Gaißert and Wallraven, 2012), it is difficult to capture the shape complexity of natural objects with parametric approaches and avoid possible confounds or special cases in object shapes (Haushofer et al., 2008; Lee Masson et al., 2016). To bridge the gap between highly familiar and novel 3D objects, we used a virtual phylogenesis (VP) algorithm to simulate the biological process and create a unique set of novel naturalistic 3D objects: the so-called digital embryos (Hauffen et al., 2012).

Although active object exploration leads to faster recognition (Harman et al., 1999), facilitates visual object learning (Tsutsui et al., 2019), and benefits the mental rotation of three-dimensional objects (James et al., 2002), visually active exploration integrates tactile cues regarding object size and texture in addition to visual information (Savini et al., 2010). To avoid such interaction, recent studies have displayed 3D objects on 2D screens or have required the experimenter to rotate the objects (Lee and Wallraven, 2013). To investigate the visual and touch senses separately and maintain this separation, the study by Gaißert et al. (2010b) used a head-mounted display (HMD) to present virtual 3D objects. Their HMD was set up in a darkened room that is isolated and greatly dissimilar to real-life conditions. In our current study, a virtual room was chosen to simulate a natural situation that resembles real-life conditions. We also used virtual reality (VR) technology to minimize tactile influences during active exploration and thus to eliminate the influence of the experimenter and tactile information in visual exploration (Van Veen et al., 1998; James et al., 2002).

Recent studies addressing the link between perceptual spaces from different sensory systems used similarity judgments (Cooke et al., 2007; Op De Beeck et al., 2008; Gaißert et al., 2011; Lee and Wallraven, 2013), as well as multidimensional scaling (MDS; Steyvers, 2002; Cooke et al., 2005a; Jaworska and Chupetlovska-Anastasova, 2009; Gaißert and Wallraven, 2012; Lee Masson et al., 2016). These studies demonstrated that visual and tactile perceptual spaces were highly congruent and that physical spaces derived from adjusting parameters in a parametric model can be reconstructed in vision, as well as in touch. Still, which complex shape features are used by both visual and tactile modalities to represent objects in the brain in similar perceptual spaces remains unknown. Further, this study assessed how a combination of shape features affects the perceived similarity between objects compared with a single shape feature.

We investigated the role of shape in reconstructing the same perception of objects in visual and tactile systems to mediate interchangeability between both modalities. To this end, we extracted computational features from the digital embryos created by our VP algorithm to assess the similarity between visual and tactile perceptual spaces. Our results show that the visual and tactile modalities not only generate two highly congruent perceptual spaces but also share the same shape features to recognize the novel object. This finding contributes to explaining why visual and tactile senses can be interchangeable.

## Material and Methods

### Participants

A total of 50 volunteers (25 female; mean age, 24.3 ± 3.7 years; all were right handed) participated in the behavioral experiments. Twenty-five of them (13 female; mean age, 23.7 ± 2.6 years) participated in the visual similarity judgment experiment, and the remaining 25 (13 female; mean age, 24.9 ± 4.6 years) participated in the tactile similarity judgment experiment. All participants reported normal or corrected-to-normal vision, normal color vision, no history of severe hand injuries, and no history of neurologic disorders. All participants were naive to the purpose of the experiment and provided informed written consent before starting the experiment. The participants received 10 €/h for their participation. The local ethics committee of the Medical Faculty of Ruhr University Bochum (No. 17–6184) approved all experiments.

### Generation of three-dimensional objects

**Figure 1.**
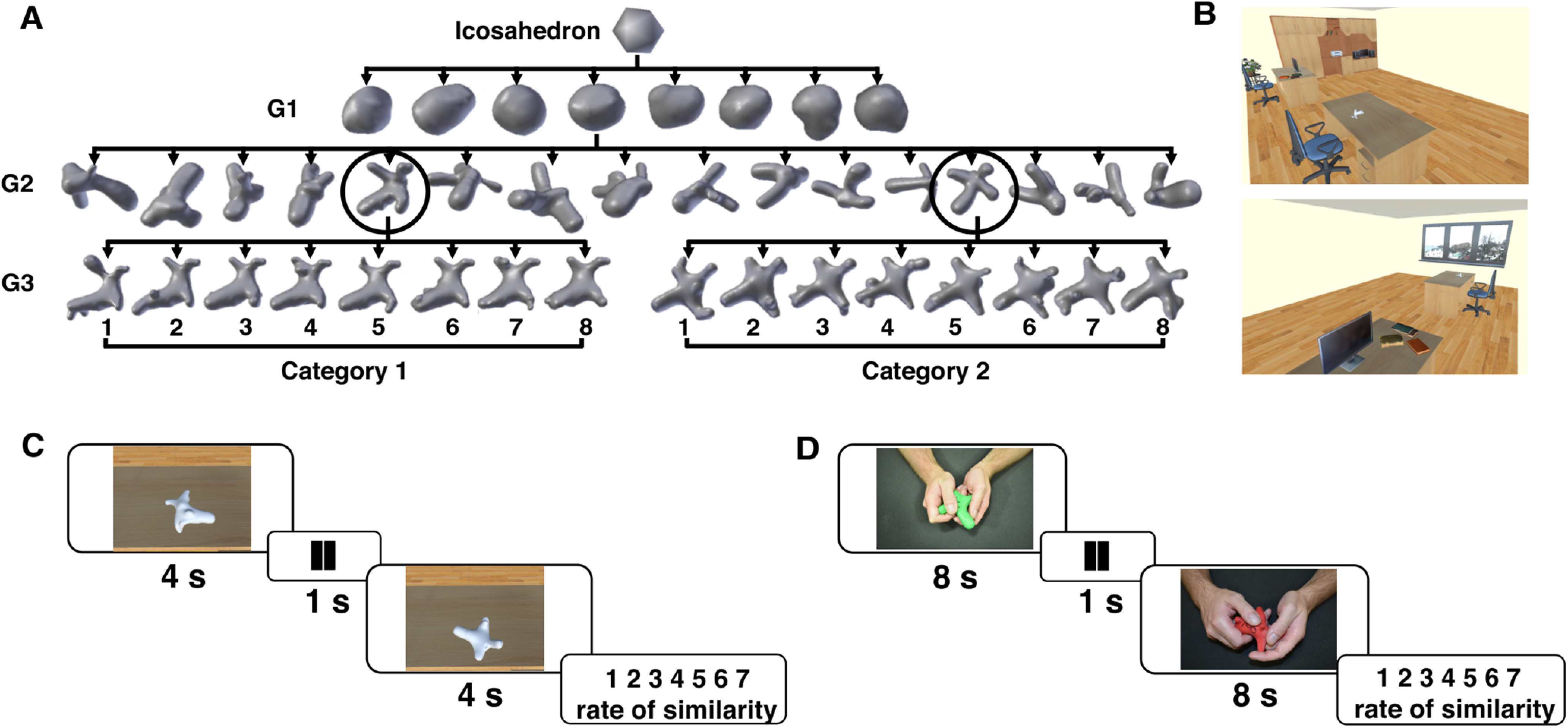
Stimuli generation and task designs. ***A***, Generating object categories using a virtual phylogenesis algorithm starting from an icosahedron. At each generation G*_n_*, selected embryos procreate, leading to generation G*_n_*_+1_. Simulated embryonic development processes were applied to a given parent object from G_2_ (circles) to generate two classes of novel objects in G_3_: eight G3 siblings from one parent formed a distinct object category. In total, two object categories from the third generation served as stimuli for the current study, with siblings 1–8 numbered by the experimenter accordingly within each category. The subjects were unaware of how the digital embryos were generated and/or categorized. ***B***, The virtual office was furnished with a desk, which was located in front of the participants. If the participants looked toward their left, bookshelves, a printer, some books, and a monitor on a study table were visible; toward their right, there was a window with a view of the outside. ***C***, Visual similarity task using virtual reality technology. ***D***, Tactile similarity experiment using 3D tangible objects generated by a 3D printer. The objects were printed out with two different colors to be more recognizable for the experimenter. Since participants were unable to see the objects, this color difference did not affect the experimental results.

To implement natural object properties and prevent evoking memories of familiar objects that might influence perceptual processing, we created naturalistic 3D objects (referred to as digital embryos) using a VP algorithm (Brady and Kersten, 2003; Hauffen et al., 2012). Digital embryos were created from a uniform icosahedron as an ancestor that was subsequently changed by simulating the biological process of embryogenesis: cell division, cell growth, and cell movement (for more details, see http://hegde.us/digital-embryos/). In the present study, 16 embryos from two categories (eight objects per category) of the third generation were selected (Fig. 1*A*). This algorithm benefits from the independent creation of shape variations within and across generated categories that are not imposed by an experimenter. In addition, the features of digital embryos are very similar to those of natural objects. Since the purpose of the current study was to identify shape features that were informative for similarity ratings between objects in both modalities, features such as weight, color, size, and material were kept constant for all objects. It is important to note that the overall appearance of objects within each category was similar (based on a pilot experiment) and that distinguishing embryos between both categories was not trivial.

In the visual experiment, we presented the stimuli in a 3D virtual reality environment to allow the participants to perform a natural exploration of objects from every possible angle without actually touching them. For this purpose, the generated digital embryos were imported into the Unity game engine 2017.2.0b8 (Unity Technologies) that delivered the 3D virtual environment wherein the similarity judgment experiment was performed. All virtual stimuli were given white matte materials.

For the tactile experiment, tangible hard plastic models of objects were printed using a 3D printer (Replicator 2X, MakerBot Industries). The printed objects were sanded with sandpaper to remove any blemish, to produce similarly smooth textures, and to deliver a similar tactile experience. The dimensions of the printed embryos were matched with those presented in the virtual environment.

### Experimental procedures

A standard similarity rating task based on a Likert-type scale was designed for both visual and tactile modalities. Participants were presented with object pairs and asked to rate the similarity between the two presented objects on a 7-point scale from completely dissimilar (1) to identical (7). As there was no definition for similarity in either experiment, the participants were required to select the features on which they based their ratings. Furthermore, the participants were not informed that there were two different object categories. A total of 136 pairs of objects [(16 × (15/2) + 16)] was presented in random order. A pilot study (eight participants [*n* = 4, visual experiment]) was conducted to determine the required object exploration time in the visual and tactile domains, as well as the necessary number of repetitions for each pair. Based on these results and previous studies that demonstrated that participants’ responses remained constant over repeated stimulus presentations (Gaißert and Wallraven, 2012), each pair was presented only once in the main experiment. In addition, during our pilot experiment, subjects needed more time to gather relevant tactile information than visual information; previous studies of cross-modal perception similarly found that tactile exploration requires twice as much time as visual exploration (Norman et al., 2004; Gaißert and Wallraven, 2012; Erdogan et al., 2015). Therefore, we decided on 4 s for visual exploration and 8 s for tactile exploration.

In both experiments, the response time required to provide a similarity rating was not restricted. Participants were instructed to use the full range of the scale during the experiment and to focus on the object features. After each pair, verbal responses were recorded by the experimenter. There were three optional breaks during both the visual and tactile experiments. Generally, the visual and tactile experiments had durations of ∼1 and 1.5 h, respectively.

After performing the task, participants completed a survey querying the object features that were important for guiding their respective similarity ratings. The listed features included (1) global shape; (2) pattern of branches; (3) number of branches; (4) size of branches; (5) global pattern; (6) concavity and convexity or curvature; (7) texture; (8) material; (9) color; and (10) weight.

### Visual experiment

The experiment was performed in a virtual office environment. The participants sat on a real chair in front of a virtual desk in a virtual office. Two perspectives in the office are presented in Figure 1*B*. The color of the walls, furniture, and lighting of the virtual room were chosen to render the details of our stimuli to be easily discernible for the participants. This virtual environment was displayed on an HTC Vive headset (www.vive.com; developed by HTC and Valve Corporation) with a resolution of 1080 × 1200 pixels/eye (2160 × 1200 pixels combined), a 110° field of view, at a 90 Hz refresh rate. By using a Vive wireless controller, participants could virtually grasp, pick up, and rotate an object freely to investigate it under different angles. The stimuli were presented at random orientations in front of the participants on a virtual desk.

Since the environment was completely new to the participants, they were familiarized with the VR environment, the proper use of the controller, and the 3D digital embryos before performing the main task. First, participants were asked to look around the virtual environment to become familiar with the virtual office and thus avoid any distractions during the main experiment. Second, participants were asked to visually examine all 16 3D digital embryos presented in random order for up to 8 s each to become familiar with their shape variations. Before the main experiment, the participants performed ten training trials that were excluded from the final analysis. In each trial of the main experiment, object pairs were presented in a random orientation in front of the participants at a fixed location on the virtual table (Fig. 1*C*). The participants were given 4 s to explore the first object. After a 1 s delay, the second object was presented for 4 s. Afterward, participants verbally reported the perceived rate of similarity (between 1 and 7) of the pair with no time restriction. Rating values were recorded by the researcher.

### Tactile experiment

Blindfolded participants were comfortably seated on a table with a sound-absorbing surface. They wore sleep masks during the entire experiment so that they had to explore the objects tactilely, without vision. As in the visual experiment, participants were familiarized with the stimulus set before the start of the main experiment and explored each stimulus for 12 s. The main task was started by performing 10 test trials that were not considered for the final analysis. Participants were allowed to freely explore and palpate objects with both hands in a natural way with no restrictions.

Each trial started with an object placed in the hands of the participant. After a start signal (a beep tone, 5 kHz, 300 ms) was played via the speaker, the exploration time started. A stop signal (same beep tone as at the start of exploration) played after 8 s indicated the end of exploration time, and the participant was required to put the object back on the table. At this moment, the experimenter replaced the first object with a second, and the exploration began following after the start signal. After 8 s, the participant was required to put the second object back on the table immediately after hearing the stop signal and rate the similarity between them verbally (Fig. 1*D*).

### Statistical analysis

#### Analysis of the similarity ratings

The ratings of all participants were averaged to obtain average similarity matrices for both the visual and tactile modalities. The correlation between the average similarity matrices of both modalities was calculated to analyze the degree of similarity between object explorations in the visual and tactile domains. Similarity matrices were converted to dissimilarity matrices by subtracting the similarity ratings from the maximum rating value of 7. To reconstruct the topology of the perceptual spaces, a nonmetric MDS implanted in MATLAB (version 2019b; Math Works) was applied to the visual and tactile group dissimilarity matrices (Cooke et al., 2007; Gaißert and Wallraven, 2012; Lee Masson et al., 2016). The MDS algorithm represents each object as a single point on a multidimensional scale. To determine the number of dimensions sufficient for explaining the data, the stress value was calculated for each dimension value from 1 to 10 (Cox and Cox, 2001; Steyvers, 2002). A statistical elbow in the stress plot indicates the number of dimensions required to represent the data. As the elbow in the stress plot represents the adequate dimension, a plateau in the squared correlation plot (RSQ) values, which are the proportion of variance of the similarity data, illustrates sufficient dimension to visualize the data (Cooke et al., 2005a, 2006). An ALSCAL MDS algorithm implanted in SPSS (IBM SPSS Statistics for Windows, version 26.0) was used to calculate the RSQ values. The weight of the first dimension was defined as the amount of RSQ for the 1D explanation to evaluate the perceptual significance of each dimension. The amount added in RSQ at a later dimension was taken as the weight for the next dimensions. As a further step, we conducted Procrustes analysis using the *Procrustes.m* function of MATLAB for both sets of points and performed a linear transformation (translation, reflection, and orthogonal rotation) to map the spaces onto each other. The resulting normalized residual sum of squared errors (*d* value*)* represents the goodness-of-fit and provides a measure of congruency between visual and tactile perceptual spaces. Because the MDS method generates relative positions in space and not absolute positions, a linear transformation is a valid operation for these kinds of data (Gaißert and Wallraven, 2012). Thereafter, to test whether the two categories of objects were represented in the brain as two distinct categories for visual and tactile modalities, the Euclidean distances between pairs of objects within each category and between pairs of objects between different categories were calculated.

#### 3D shape features extraction and selection

According to our stimulus generation algorithm and questionnaires, shape features play an essential role in rating the similarity between object pairs. Thus, in our second analysis, we investigated which object features were used to create compatible perceptual spaces for the visual and tactile sensory modalities. First, all 16 digital embryos were aligned to a similar orientation. The iterative closest point (ICP) algorithm is applied to align objects. The ICP algorithm iteratively applies transformations (a combination of translation and rotation) to minimize square errors between corresponding objects (Chen and Medioni, 1991; Besl and McKay, 1992). For further analysis, we extracted 17 relevant shape features from all 16 aligned digital embryos (Table 1). The Euclidean distance between the pairs of objects for each shape feature was calculated to generate the computational dissimilarity measures.

**Table 1:** Summary of the extracted features

Feature	Definition	Visual modality	Tactile modality
F1	Gaussian curvatures of objects	✓	×
F2	The distances from all vertices to the left of objects	✓	✓
F3	The surface area of objects	×	×
F4	The volume of objects	✓	✓
F5	The area of the projection of the object to *x–y*-plane (top/back view)	✓	✓
F6	The area of the projection of the object to *x–y*-plane (lateral view)	×	×
F7	The area of the projection of the object to *x–y*-plane (frontal view)	✓	×
F8	The distances of the left from edges on the *x–y*-projection	✓	×
F9	The distances of the left from edges on the *x–y*-projection	×	×
F10	The distances of the left from edges on the *x–y*-projection	×	×
F11	Geometric measure: bounding box size	✓	✓
F12	Geometric measure: bounding box diagonal	×	×
F13	Geometric measure: inertia tensor	✓	✓
F14	Geometric measure: principal axes	✓	×
F15	Geometric measure: axis momenta	✓	✓
F16	The left of objects	×	×
F17	Topological measure: the number of faces that constructed objects	×	✓

The first and second columns illustrate 17 extracted shape features. The third column represents the selected feature that demonstrates the lowest *d* value between the physical and the visual perceptual spaces. The fourth column shows the selected features that lead to a minimum *d* value between physical and the tactile perceptual space.

In line with the results of the questionnaires, one important feature was the curvature of the objects. Because the objects are basically meshes formed by triangles, the Gaussian curvature (F1) at each vertex was calculated by computing the curvature tensor and the principal curvatures at each vertex of a digital embryo (Shum et al., 1996; Rusinkiewicz, 2004). Differences between the curvatures of the vertex of each mesh for all pairs of objects were calculated to perform a shape dissimilarity matrix based on curvature. The Euclidean distances from all vertices to the center of an object (F2) provide additional information about the global curvatures. The surface area (F3) and volume (F4) of an object indicate its size. The different views of an object contain sufficient information about the shape (F5–F10). 2D projections provide 2D perspectives of the variance of an object. The 2D projection to the *x–y-*, *y–z-*, and *x–z*-planes gives a perspective view from the top, lateral, and frontal sides of the object, respectively. Geometric measures represent the geometric properties of an object, such as size, shape, angle, position, and dimension. The size and diagonal of the smallest enclosing bounding box illustrate the size, volume, pattern of branches, and number of branches (F11*–*F12).

The mass distribution provides information about the patterning of the branches of the objects. One possible way to describe the mass distribution in a rigid body is the inertia tensor (F13). Previous studies have identified the informational value of the inertia tensor in the tactile perception of object properties (Pagano et al., 1994; Carello et al., 1996; Kingma et al., 2002; Cabe, 2019). The eigenvalues of the inertia tensor or principal axis (F14) have been shown to be related to the perception of an object’s shape, length, width, height, and heaviness. The eigenvectors of the inertia tensor or moments of inertia tensor (F15) are related to the perception of an object’s orientation and grasp position (Kingma et al., 2002). To analyze all geometric measures, we used the 3D mesh processing system Meshlab_64bit (https://www.meshlab.net/; Cignoni et al., 2008). Furthermore, we used the number of triangles (F17) that formed the digital embryos as a relevant feature for the visual and tactile systems. Based on the process of the algorithm for generating digital embryos, when fission proceeds, a triangle is split into four new triangles, indicating that the number of vertices increases. If these vertices are independent, they will move about in space according to the force applied to them. From this, we concluded that more vertices leads to more tiny concavity and convexity on the objects. These tiny bumps could refer to surface quality or texture, and they are comprehensible for tactile sense and even for visual sense.

To evaluate each single feature validity, we defined two criteria. The first criterion was defined as the mean *d* value in fitting the physical map derived from each feature to all individual subject maps (single-fitting error). The second criterion was the mean *d* value in fitting each individual map to all other individual maps (cross-fitting error). Cross-fitting error defines how well individual subject maps are fit to each other. If the single-fitting error generated by each feature and the cross-fitting error are not significantly different, it could be considered that the feature fits the human data well (Cooke et al., 2005a, 2006). To test perceptual validity, we performed a two-tailed *t* test between single-fitting and cross-fitting errors (corrected for multiple comparison).

Further, given that a combination of different features reconstructs a different perceptual space, and in this study, the combination of features plays a role, we performed perceptual validity analysis on the combination of features as well. We evaluated all combinations of features listed in Table 2, comparing the mean *d* value in fitting a physical map derived from each combination to all individual maps (combination fitting error) with the mean *d* value in fitting each individual map to all other individual maps (cross-fitting error).

**Table 2:** The goodness of fit

*N*	Minimum *d* values for *N* combination of features (*N* = 1–17)	
	Fit quality between physical and visual perceptual spaces	Fit quality between physical and tactile perceptual spaces
1	*d* = 0.158 (F13)	*d* = 0.266 (F13)
2	*d* = 0.120 (F2, F13)	*d* = 0.215 (F2, F9)
3	*d* = 0.098 (F5, F13, F14)	*d* = 0.158 (F2, F4, F13)
4	*d* = 0.072 (F2, F11, F13, F14)	*d* = 0.135 (F2, F4, F11, F16)
5	*d* = 0.061 (F2, F5, F11, F13, F14)	*d* = 0.126 (F2, F4, F9, F15, F17)
6	*d* = 0.062 (F2, F3, F6, F12, F14, F15)	*d* = 0.118 (F2, F4, F5, F10, F11, F17)
7	*d* = 0.053 (F2, F5, F6, F11, F13, F14, F15)	*d* = 0.101 (F2, F4, F5, F11, F13, F15, F17)
8	*d* = 0.052 (F2, F3, F4, F8, F11, F13, F14, F15)	*d* = 0.110 (F2, F4, F5, F6, F9, F10, F11, F15)
9	*d* = 0.050 (F2, F4, F5, F6, F7, F11, F13, F14, F15)	*d* = 0.116 (F2, F4, F7, F10, F11, F12, F13, F15, F16)
10	*d* = 0.048 (F1, F2, F4, F5, F7, F8, F11, F13, F14, F15)	*d* = 0.120 (F2, F4, F5, F7, F9, F10, F11, F14, F15, F17)
11	*d* = 0.050 (F2, F4, F5, F6, F7, F10, F11, F12, F13, F14, F15)	*d* = 0.132 (F2, F3, F4, F7, F9, F10, F11, F12, F14, F15, F16)
12	*d* = 0.060 (F1, F2, F4, F5, F7, F8, F10, F11, F12, F13, F14, F15)	*d* = 0.145 (F2, F3, F4, F6, F7, F9, F10, F11, F12, F14, F15, F17)
13	*d* = 0.078; all features were selected except F6, F9, F12, F17	*d* = 0.150; all features were selected except F1, F6, F8, F13
14	*d* = 0.110; all features were selected except F6, F16, F17	*d* = 0.171; all features were selected except F5, F8, F13
15	*d* = 0.167; all features were selected except F10, F14	*d* = 0.271; all features were selected except F10, F14
16	*d* = 0.168; all features were selected except F14	*d* = 0.272; all features were selected except F14
17	*d* = 0.168; all features were selected	*d* = 0.272; all features were selected

The d values in the columns represent the minimum *d* values between the physical and the visual/tactile perceptual space for a different combination of features. The best fit quality between physical and visual perceptual spaces occurred when the ten features F1, F2, F4, F5, F7, F8, F11, F13, F14, F15 were selected. On the other hand, the combination of the seven features F2, F4, F5, F11, F13, F15, F17 lead to the best fit quality between physical and tactile perceptual spaces. These two modalities share the features F2, F4, F5, F11, F13, F15. (Extended Data Table 2-1).

10.1523/ENEURO.0101-21.2021.t2-1 Table 2-1 The *d* values in Table 2 show that visual and tactile *d* values lead to U-shaped curves. A single feature or a combination of a few features led to high *d* values, and when the number of involving features rose, the *d* values again increased. It supports the notion that humans do not necessarily need to use all given features to reconstruct the perceptual spaces. Download Table 2-1, TIF file.

To assess which combination of features forms highly similar veridical spaces in human visual and tactile perception, different combinations of features were tested to create different physical spaces. There are 131,071 different combinations, ranging from a single feature to combinations of 17 features (_17_*C*_1_+_17_*C*_2_+_17_*C*_3_+…+_17_*C*_16_+_17_*C*_17_ = 217 – 1). The physical space for each combination was created by applying an MDS analysis to the dissimilarity matrix obtained from the pairwise distances of features. For example, there are 136 (_17_*C*_2_) different combinations of two features (F1–F2, F1–F3, …, F16–F17). After normalizing the pairwise distance of each feature, an MDS analysis was applied to the average of two normalized distances of the corresponding features to form physical spaces. To evaluate the validity of the perceptual space, the goodness-of-fit criterion (*d* value) as a linear transformation (translation, reflection, and orthogonal rotation) was applied to assess the map fitting between physical spaces and the visual and tactile perceptual space. A feature combination of F2 and F13 resulted in highly similar physical and visual perceptual spaces (i.e., the best fit of the visual perceptual spaces to the physical space was achieved using the combination of the distances from all vertices to the center of the object and its inertia tensor). We repeated these steps for both modalities to calculate the *d* values for all possible feature combinations.

## Results

### Visual and tactile perceptual spaces

**Figure 2.**
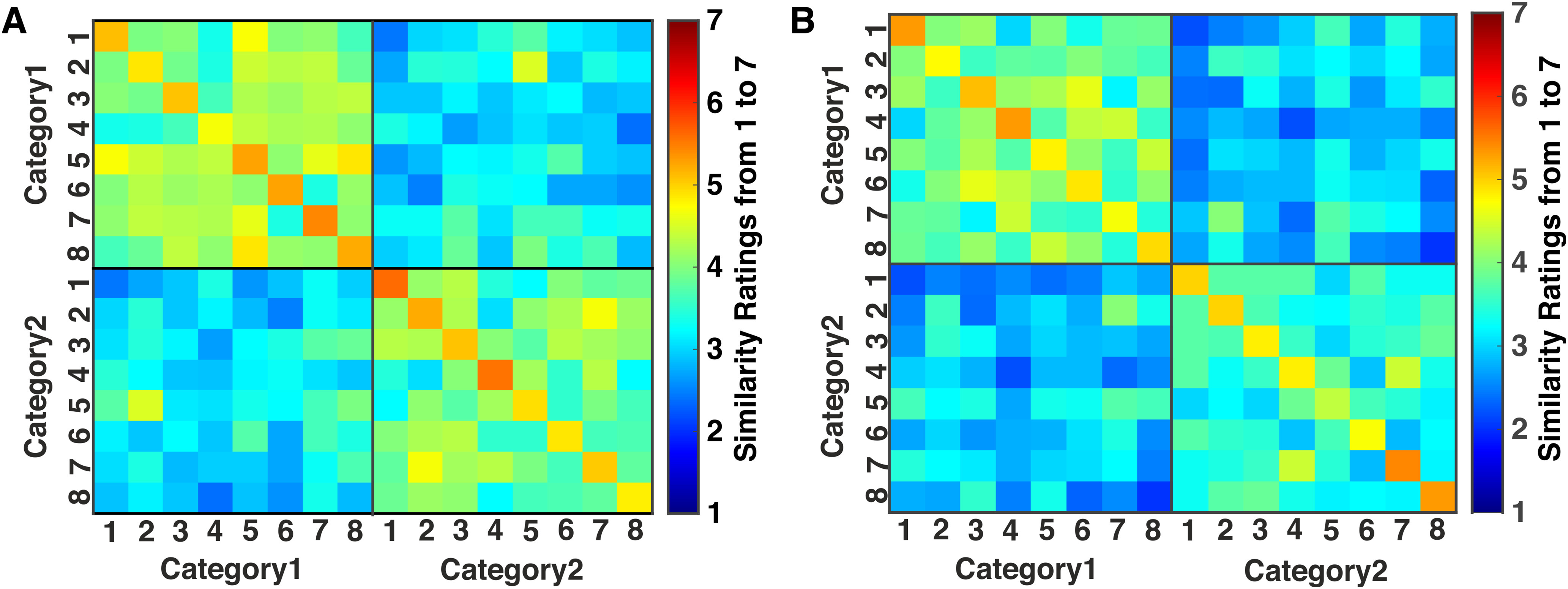
Similarity matrices. ***A***, Average group similarity matrix for visual similarity judgment. ***B***, Average group similarity matrix for tactile similarity judgment. The color codes for the similarity ratings corresponded to the numbers, ranging from 1 (dissimilar, dark blue) to 7 (identical, dark red). Numbers on the *x*- and *y*-axes refer to the digital embryos in each category (eight objects per category) according to Figure 1*A*.

**Figure 3.**
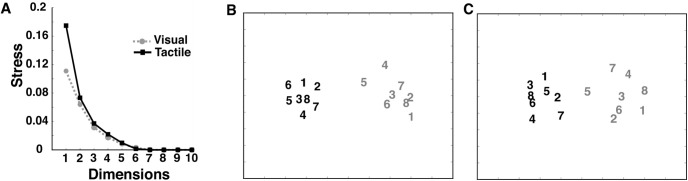
Two-dimensional visual and tactile perceptual spaces. ***A***, The stress values for both modalities were calculated for 1–10 dimensions. The elbow indicates that two data dimensions are sufficient to explain the visual and the tactile perceptual space. ***B***, Two-dimensional visual perceptual space (Extended Data Fig. 3-1*A*,*C*, one- and three-dimensional visual perceptual spaces). ***C***, Two-dimensional tactile perceptual space (Extended Data Fig. 3-1*B*,*D*, one- and three-dimensional tactile perceptual spaces). The numbers refer to the object numbers in each category according to Figure 1*A*. Contrast level codes for different categories; black, category 1; gray, category 2.

One group of 25 participants underwent the similarity judgment experiment in the visual modality, while another participated in the tactile modality. The average similarity matrices across all participants are shown in Figure 2. We observed a high correlation (*r* = 0.82; *p* < 0.001) between the visual and tactile similarity matrices, which indicates an equal interpretation of object similarities for both modalities. Using the average dissimilarity matrices, we ran an MDS analysis to calculate the stress values for 1–10 dimensions for both modalities (Fig. 3*A*).

10.1523/ENEURO.0101-21.2021.f3-1 Figure 3-1 One- and three-dimensional visual and tactile perceptual spaces. ***A***, One-dimensional visual perceptual space. ***B***, One-dimensional tactile perceptual space. ***C***, Three-dimensional visual perceptual space. ***D***, Three-dimensional tactile perceptual space. The numbers refer to the object. Color codes two different categories. Download Figure 3-1, TIF file.

To select the number of sufficient dimensions for our similarity data, we applied the statistical elbow method. Because human data mostly contain noise, stress values of zero are not observed in empirical data; moreover, the lower the stress value, the higher the data dimensionality. Several studies have shown that a stress value of <0.2 is sufficient to describe human data faithfully (Clarke and Warwick, 2001; Cooke et al., 2007; Gaißert et al., 2008). The elbow in the stress plot was visible in two or three dimensions (Fig. 3*A*). Given that the stress values for all dimensions were <0.2, one dimension was also sufficient to visualize perceptual spaces, although the elbow in the stress plot was visible in two or three dimensions. Furthermore, the mean weight for the first dimension across visual group was 0.958, while the mean weight of the second and the third dimension were 0.018 and 0.015, respectively. Similarly, in the tactile group, the weight of the first dimension was 0.897, and the mean weights of the second and the third dimensions were 0.0629 and 0.0256. These results not only prove again the higher importance of the first dimension for the visual and tactile modality, but also demonstrate that the second and third dimensions play a minor role in the data interpretation. Here, for better visualization, we plotted the visual and tactile perceptual spaces for two dimensions, although the second dimension was of little importance in information reconstruction. The MDS output for two dimensions for both modalities (Fig. 3*B*,*C*, Extended Data Fig. 3-1) showed highly similar perceptual spaces across the visual and tactile modalities (*d* = 0.136; zero indicates perfect alignment). These results indicate that, in the absence of visual perception, tactile inspection is capable of reconstructing the same perceptual space as the visual system, and vice versa, even for unfamiliar objects.

**Figure 4.**
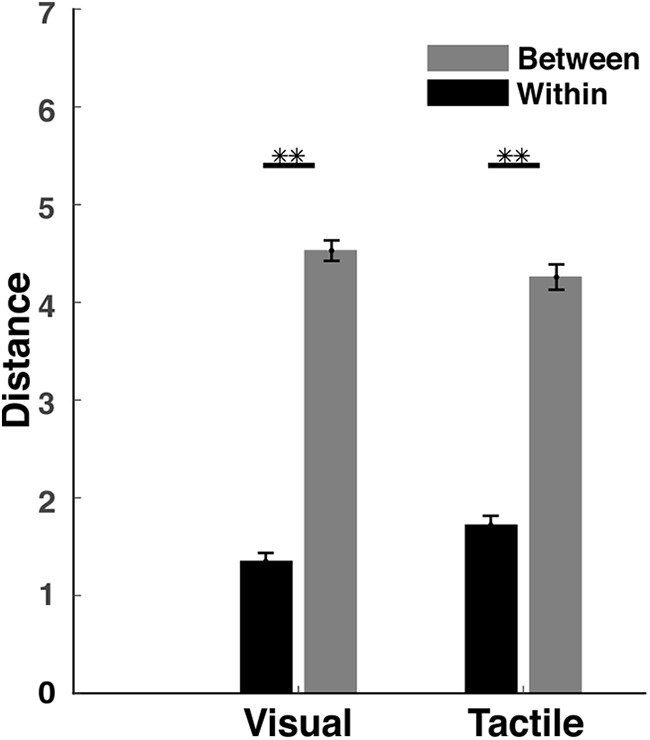
Euclidian distance. The average distance between pairs of objects within a category (black bars) is significantly smaller than the distance between pairs of objects from different categories (gray bars) for both modalities. Error bars represent the SEM. **p < 0.0001.

In addition to the highly congruent perceptual spaces of visual and tactile exploration, the two clusters represent the two object categories of the VP algorithm. Here it is important to point out that the participants were not aware of any of the categories to which the objects belonged. For further analysis, we investigated the degree of cluster definition in both perceptual spaces by measuring the Euclidean distances between pairs of objects within a category and pairs of objects between categories. The results indicate a significant difference between within-category distances and between-category distances (visual: *t*_(118)_ = 23.1, *p* < 0.0001; tactile: *t*_(118)_ = 15.4, *p* < 0.0001; Fig. 4).

### Perceptual validation of computational features

The observed similarity between visual and tactile perceptual spaces raises the question of which stimulus features contribute to the formation of these highly congruent perceptual spaces.

**Figure 5.**
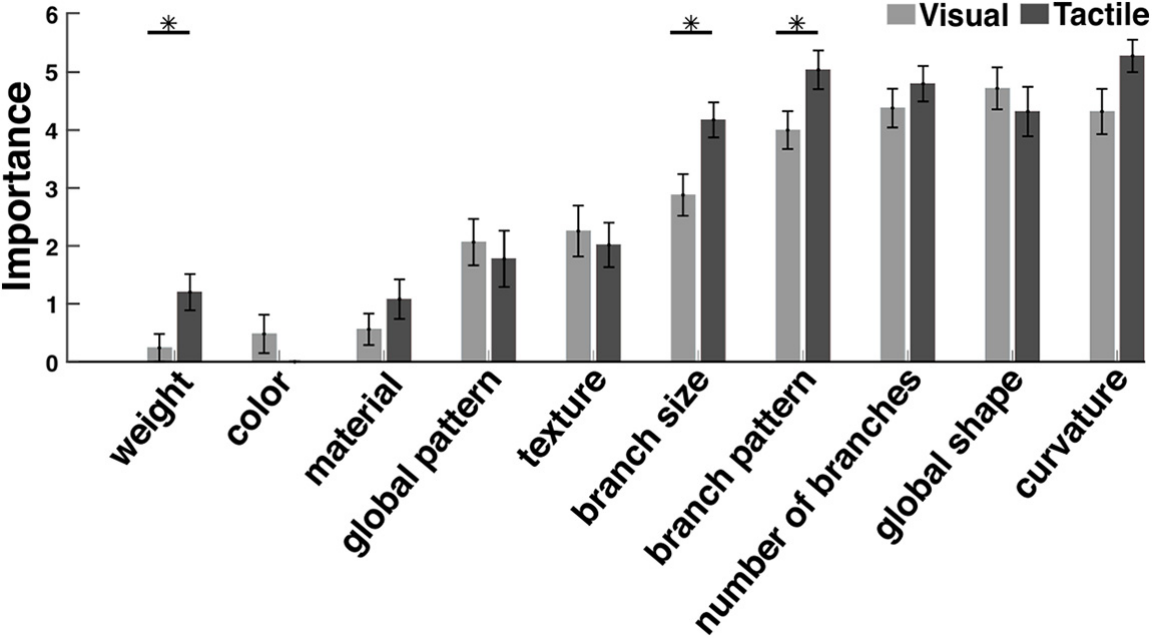
Questionnaires. At the end of each similarity judgment test, participants were asked to rate the importance of features to determine which features played the main role in their similarity judgments. In addition to the weight, color, material, global patterning, and texture, we listed further details describing the shape of digital embryos: branch size, branch pattern, number of branches, global shape, and curvature. The results for both modalities indicated that the shape features played a major role. Features such as weight, size, and the pattern of branch distributions were significantly more important for the tactile similarity judgment experiment than for the visual. Bars represent the mean ratings across all participants over the visual (gray) and tactile (black) modalities (0 means no importance; 6 means very important). Error bars represent the SEM. *p < 0.01.

The average volume of objects within categories 1 and 2 were 8.02 ± 0.45 × 8.65 ± 0.24 × 5.39 ± 0.21 cm^3^ and 8.21 ± 0.32 × 8.76 ± 0.48 × 5.44 ± 0.20 cm^3^, respectively. There was no significant difference in the length (*t*_(7)_ = 0.9167, *p* = 0.3898), width (*t*_(7)_ = 0.4489, *p* = 0.6671), or height (*t*_(7)_ = 0.5706, *p* = 0.5861) of objects between the two categories. The average weight of objects within categories 1 and 2 were 13.62 ± 0.52 and 13.5 ± 0.53 g, respectively. There was no significant difference between the weight of objects in both categories (*t*_(7)_ = 0.4237, *p* = 0.6845). Therefore, object features such as weight and size do not contribute to object categorization. In contrast, shape features, such as the number of branches, size, and pattern, play a major role in object categorization, as shown by our questionnaire results (Fig. 5).

As can be seen in Table 2, visual and tactile modalities share the following six common features: the distances from all vertices to the center of objects (F2); the volume of objects (F4); the area of the projection of the object to *x–y*-plane (F5); bounding box size (F11); inertia tensor (F13); and axis momenta (F15). Referring to the surface texture of the objects, the number of triangles (F17) plays a major role in the tactile modality. Four features exclusive to the visual modality include Gaussian curvatures of objects (F1), the area of the projection of the object to the *x–z*-plane (F7), the distances of the center from edges on the *x–y*-projection (F8), and the principal axes (F14).

To find relevant features, we calculated the physical space for each possible feature combination (1-17 features) using the MDS method. The goodness of fit criterion (*d* value) was calculated between all physical spaces and the visual or tactile perceptual spaces (i.e., the higher the fit, the lower the *d* value). The minimum *d* values are listed in Table 2.

**Figure 6.**
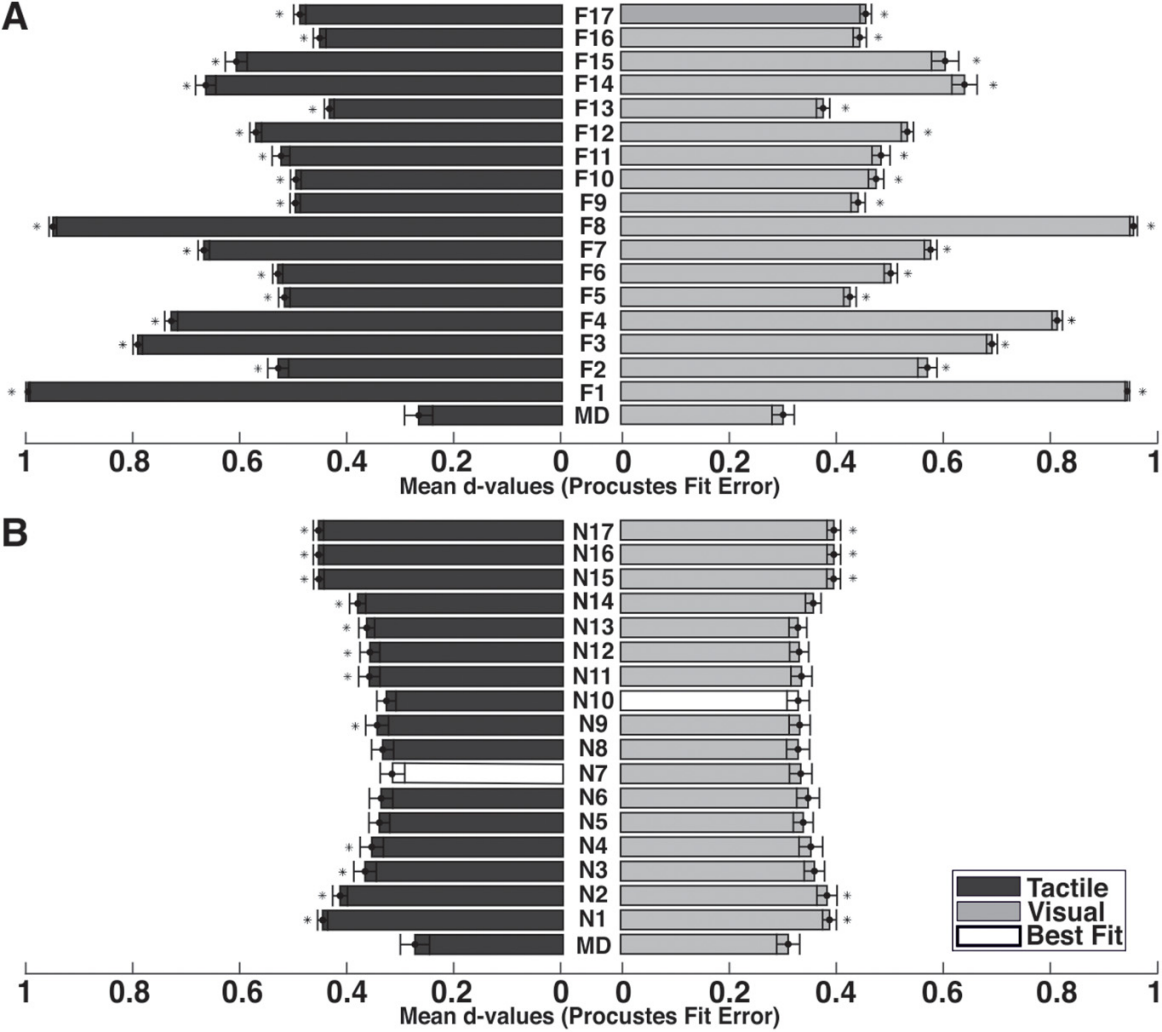
Mean *d* values (Procrustes fit error). ***A***, Fits between computational measures of a single feature with visual and tactile maps. ***B***, Fits between computational measures of a combination of features with visual and tactile maps. F1–F17 indicate the number of single features. N1–N17 present a combination of features that are listed in Table 2. Asterisks demonstrate that a reconstructed map of a feature or combination of features is significantly different from perception in human behavior (*p* < 0.01). MD indicates cross-fitting error.

In fitting to the human visual/tactile map, the single-fitting error differed significantly from the mean cross-fitting error. Note that, although single-fitting errors provided a poor fit (*p* < 0.001; Fig. 6*A*), combination fitting errors provided better fits (Fig. 6*B*). Our results indicate that some features of an object may not be meaningful on their own, but their combination creates a meaningful feature that leads to the correct perception of an object. There are several feature combinations that are statistically close to human perceptual maps. Since these combinations share several common features, we decided to focus only on the combination of features which caused the absolute minimum *d* value to make the comparison between visual and tactical modalities possible (see Discussion).

A physical space derived from a combination of 10 features showed the best similar fit (minimum *d* value, *d* = 0.048) to the mean perceptual space derived from the human visual system. Moreover, comparing physical spaces derived from a combination of features to the stimulus space derived from human tactile perception showed that a combination of seven features constructed a physical space with the minimum *d* value (*d* = 0.101). These findings demonstrate that participants use multiple features rather than single features in their perceptions. On the other hand, visual and tactile sensory systems share common features in object identification.

## Discussion

The current study addressed the role of shape features that mediate the interchangeability between visual and tactile modalities. To this end, we generated a set of complex, natural digital embryos (3D objects) based on a VP algorithm that simulates the biological process of embryogenesis. These 3D objects were used to perform similarity rating experiments using visual and tactile modalities. One objective of the current study was to provide a more realistic situation in which participants were confronted with the perception of 3D objects, while minimizing tactile influences during active visual exploration and eliminating the influence of the experimenter. Moreover, neuroimaging studies demonstrated that the cortical mechanisms of three-dimensional (3D) shape processing are different in vision and touch, while cortical mechanisms of two-dimensional form are similar (Hsiao, 2008). Hence, to implement the goals mentioned, the visual experiment used VR technology to enable active and unconstrained visual exploration without additional tactile information. In the tactile experiment, subjects explored the same objects as 3D plastic printouts while being blindfolded. Overall, we showed highly congruent visual and tactile perceptual spaces that are most likely based on shared common features between the spaces.

Seminal studies demonstrate that tactile and visual sensory systems are both accurate in shape discrimination. Visual perception is based on parallel processing of transforming light from our 3D environment into a two-dimensional retinal image, while tactile perception is serially developed through an exploratory procedure on 3D objects (Gaißert et al., 2010a; Klatzky and Lederman, 2011). In our study, object differences were computed using 17 2D and 3D features. Curvature is one of the important features that has been shown play an important role for visual and tactile perceptions (Pont et al., 1997; Barth et al., 1998; Lim and Leek, 2012). Curvature information plays a particularly important role to provide the three-dimensional information of the surface structure of an object (Todd, 2004; Strother et al., 2015; Yue et al., 2020). fMRI studies of monkeys and humans demonstrated that the temporal cortex and retinotopic regions of the visual system are involved in the processing of curvature information (Yue et al., 2020). However, curvature information is processed by cutaneous receptors in the tactile system (Kappers, 2011) to judge the differences between objects. Furthermore, the front and back views of objects are more informative in the visual and touch systems, respectively (Newell et al., 2001). Our results revealed that frontal and top/back views played a role in visual exploration and top/back views played a role in tactile exploration, consistent with findings by Newell et al. (2001). In addition, the mass, center of mass, and the inertia tensor are an important set of physical properties for visual and tactile perceptions. They describe the mass distribution, object’s weight, and resistance to motion changes during viewing or manipulating an object. Humans are able to perceive elements of the inertia tensor of the held objects through dynamic touch, which is a simulation mechanism of muscular sensitivity to the inertia parameters (Fitzpatrick et al., 1994; Casati and Pasquinelli, 2005; Mavrakis and Stolkin, 2020).

Among these common features, participants relied on inertia tensor information during both tactile exploration and visual inspection. While several studies have proven the role of inertia tensor in tactile perception (Pagano et al., 1994; Carello et al., 1996; Kingma et al., 2002; Cabe, 2019), the role of proprioceptive features in visual perception is unclear. The integration of perception with active exploration, however, offers a possible explanation: when participants explore an object in a VR environment using a controller, they are able to change the object orientation without any restriction to view objects from all sides; hence, they can collect inertia information, such as the length, width, height, and mass distribution of the object, while rotating their wrists to control the orientation of objects. Together, our results suggest that the inertia tensor plays a role in visual and tactile perception if humans explore objects in a natural, active manner for similarity judgments and object identification. In addition, global features related to the size and volume (F2, F4, and F11) contributed to both modalities: by viewing and grasping objects with the hands, these features can be obtained from any object. Furthermore, the top view of the objects (F5) contained relevant information for both the visual and tactile senses. Because the top view of objects presents the largest surface area, it may provide more information about the shapes of objects.

In contrast, some features were exclusive to the visual or tactile system. The Gaussian curvature feature (F1), which describes the convexity and concavity of an object at the vertices, only played a role in visual perception. This finding may be attributed to the following: when humans explore an object, the overall convexness and concaveness of the object can be perceived literally at first glance by the visual system, whereas the fingertips gather only limited curvature information related to those parts of an object that are actually touched; therefore, obtaining curvature features by touch requires a much higher sample rate. On the contrary, as a feature that only contributes to the tactile modality, the number of triangles contained in the 3D objects describes the surface roughness (i.e., its texture). Texture and shape are of equal importance during tactile conditions (Cooke et al., 2005b, 2007). Notably, cell division during embryo generation caused tiny variations in object texture, which may be easily recognized by the tactile system. We sanded the surfaces of all plastic objects to minimize this effect but, given the importance of texture in the tactile modality, even the smallest deviations between objects may provide relevant information. Cooke et al. (2005a,b, 2006) established a high-level approach to validate the extracted physical features by comparing behavioral perceptual spaces with physical spaces derived from computational measures. They extracted six features in the first study and eight features in the second. Half of the features were 2D features and were related to gray values between objects. Other features were extracted from a 3D mesh, such as object perimeter and curvature. In general, they tested single features to validate the physical space of a computational measure. However, in real life the combination of several features guides human object recognition. For instance, to identify a walnut, a pecan, a plum, and a cherry, relying only on the perimeter helps in identifying the cherry. If we pay attention to bumpiness, softness, and perimeter simultaneously, it is possible to identify all of these objects accurately. To close this gap, in the current study, 17 features from a 3D mesh were extracted. We extracted 17 features from different modalities to better describe the physical properties of objects using both visual and tactile modalities. However, in our study, features were extracted from 3D mesh, not from 2D photographs. The extracted features describe the three-dimensional nature of objects and are similarly comprehensible for both visual and tactile senses. These features mainly describe the shape of objects. Most importantly, high-level and abstract features that participants are not capable of describing easily have been ignored. In addition to single-feature comparisons, we used different feature combinations to detect the optimal combination to describe the perceptual space of various modalities.

Several feature combinations play a role in reconstructing human perceptual spaces in visual and tactile modalities. Although we focused only on the feature combination that caused the absolute minimum *d* value, selecting other feature combinations in the same range would not contradict the finding of the current research. With a brief reflection on the results (Fig. 6, Table 2), it is obvious that even nonsignificant feature combination maps share common features that can reconstruct visual and tactile human perceptual maps. For instance, the distances from all vertices to the center of objects (F2) is an important feature that plays a major role in visual and tactile representation, regardless of which combination is chosen. This feature provides some information about global shape, mass distribution, and pattern of branches. The surface area (F3), the volume of objects (F4), and bounding box size (F11) are other features that are mostly involved in both modalities. These features are recognizable by both senses, and they describe shape objects as well. The top/back view of the objects (F5) is also a relevant feature that in combination with other features leads to the perception and identification of objects. Inertia tensor (F13–F15) as a physical–mechanical description of object properties is associated with the perception of the shape, length, width, height, heaviness, orientation, and grasp position of an object. To choose the same criterion for comparing the visual and tactile spaces, considering that a low *d* value close to zero indicates a better fit, we chose the combination with the absolute minimum *d* value in both modalities to represent human perceptual maps.

Overall, our findings raise the question of how the brain uses shape information from two different modalities to form highly congruent perceptual spaces. Conceivably, the brain can form a multimodal perceptual space for relevant features, which is the object shape. This multimodal perceptual space is accessible to both modalities. For example, if a person is trained to categorize objects based on shape variations either visually or tactually, he or she can categorize novel objects using visual or tactile information even in the absence of trained sensory cues (Yildirim and Jacobs, 2013; Wallraven et al., 2014). This is presumably because a shared multisensory representation integrates the sensory information of the shape independent of the input modality. Corroborating this assumption, many neuroimaging studies on multisensory perception emphasize a common neural substrate in visual and tactile shape processing (Amedi et al., 2001; Op De Beeck et al., 2008; Drucker and Aguirre, 2009; Lee Masson et al., 2016). Amedi et al. (2001) found a region within the human lateral occipital complex (LOC) that is activated during multimodal object perception. More recently, Lee Masson et al. (2016) showed that the lateral occipital cortex, as a multisensory convergence area, becomes activated during visual and tactile shape processing. These findings implicate the LOC as a candidate region to encode the multimodal perceptual space of shape processing independent of modalities (Lacey et al., 2009). The existence of such a multimodal perceptual mechanism might be the main reason why humans can interchangeably use visual and tactile modalities because the acquired object information can be shared or transferred between modalities.

The sharing of certain common features among sensory inputs is a prerequisite for the integration of sensory information from different modalities (Holmes et al., 2008). Physically, the adequate stimuli and perception of photoreceptors and mechanoreceptors differ significantly from each other. Nevertheless, they both provide detailed and congruent information about the perceptual space. Learning, in particular categorization, can have a strong influence on the dimensionalization of complex objects (Palmeri et al., 2004). Although previous studies used a parametrically defined complex object space to determine whether the tactile and visual modalities are capable of forming a veridical perceptual space (Gaißert et al., 2008, 2010b; Lee Masson et al., 2016); the brain does not necessarily need to use all given dimensions (i.e., object features) to represent the perceptual spaces. The visual system might rely on a limited number of independent shape features to distinguish the shape of objects between categories (Ullman et al., 2002; Op de Beeck et al., 2003). During visual object exploration, only the relevant features are enhanced, and irrelevant features are suppressed. Because the tactile perceptual system has much in common with the visual system (Cooke et al., 2007; Gaißert et al., 2008, 2010a, 2011; Lacey and Sathian, 2014), it can be assumed that shape exploration in the tactile system also uses only a limited number of dimensions. In our investigation, different combinations of extracted features were used to find informative shape features that constructed two veridical physical spaces akin to the visual and tactile perceptual spaces. Our results indicate that a combination of 10 features forms a physical space with maximal similarity to the visual perceptual space, whereas a combination of seven features was capable of describing a physical space that is highly similar to the tactile perceptual space. Based on the *d* values in Table 2, the plot of *d* values of all possible combinations when fitting the visual and tactile perceptual spaces with the physical map demonstrates a U-shaped curve (Extended Data Table 2-1). This U-shaped curve demonstrated that a single feature/a combination of a few features led to high *d* values, and when the number of involved features rose, the *d* values again increased. This is consistent with the notion that not only do humans use multiple rather than single features in their perceptions, but also that they do not necessarily need to use all given dimensions (i.e., object features) to represent the perceptual spaces (Ullman et al., 2002; Cooke et al., 2007; Lacey et al., 2014). This finding shows that relying on a set of fixed dimensions might facilitate the transfer of knowledge across modalities.

Categorization is an essential ability of the human brain, as it enables the organism to interact with its surroundings in such a way as to ensure survival in a dangerous environment. While several models describe human categorization behavior (e.g., the prototype theory, exemplar theory, and decision bound theory; Ashby and Maddox, 2011), they all share a common feature between them: namely, the similarity of objects. Therefore, similarity is a key component in identifying and categorizing new objects (Gaißert et al., 2011). In our current study, perceptual spaces revealed clear object clusters based on similarity, despite the participants having no prior knowledge of the categories of the objects. Shepard (2001) have proposed that objects from the same category should be locally close in perceptual spaces. Our results reveal that clusters within perceptual spaces correspond to different object categories. Furthermore, research by Edelman (1999) claimed that objects from the same category should be represented in perceptual spaces within a single cluster. Our results demonstrated that the distance was lower for pairs of objects with greater similarity (i.e., same category) and higher for pairs of objects with less similarity (i.e., different categories; Fig. 4). The role of similarity in forming the basis of perceptual categorization and the role of shape in the formation of category structure have been controversial for a long time among cognitive neuroscientists (Moore, 2002). Our results support previous findings that similarity plays an important role in categorization.

However, in our study, a VP algorithm was used to create a unique set of novel, naturalistic 3D objects and to avoid possible confounds or special cases in object shapes. This algorithm benefits from the independent creation of shape variations within and across generated categories that were not imposed by an experimenter. In addition, the features of digital embryos are very similar to those of natural objects. While these stimuli provide a rich set of objects with which to investigate the scientific questions, there is no underlying parameter space to identify and control the dimensionality of digital embryos. This limits restoring the dimensionality of the physical objects from the MDS output. Given that the purpose of the current study was to create naturalistic object categories that differ only in shape, the VP algorithm made it possible to design our desired objects.

**Figure 7.**
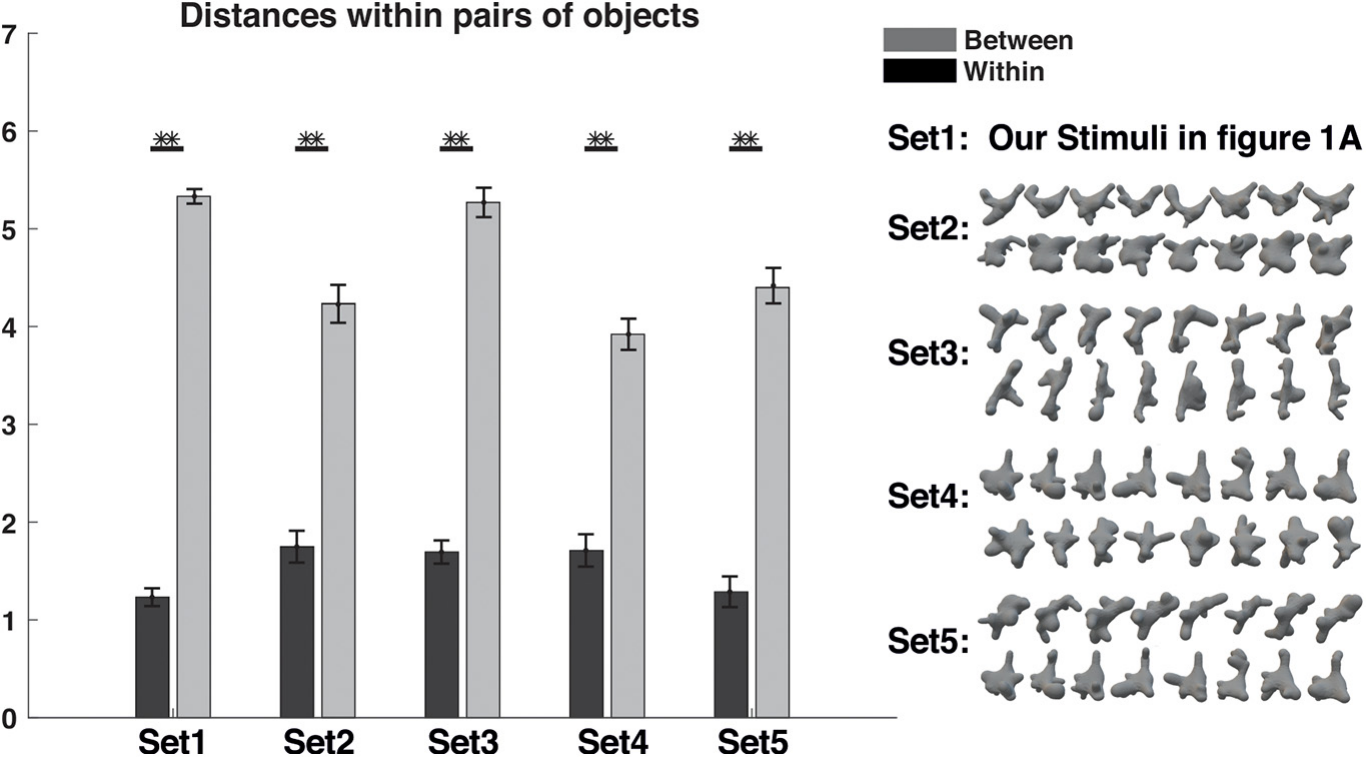
The average distance for five different sets. The average distances between pairs of objects within and between pairs of objects for five different sets of objects. Within category, black bars; between categories, gray bars. Error bars represent the SEM. **p < 0.0001.

Further, to demonstrate that our methods are reliable on other stimuli sets, four different random category sets were created using a VP algorithm. Figure 7 reveals that our methods were able to discriminate all different categories well.

Together, our results indicate a link between perceptual spaces of visual and tactile systems, which suggests that both modalities use a similar cognitive process to represent shape information. Elucidating these interactions between modalities could help to advance understanding of how humans can interchangeably use different modalities to interact with their surroundings.

## Synthesis

Reviewing Editor: Satu Palva, University of Helsinki Decisions are customarily a result of the Reviewing Editor and the peer reviewers coming together and discussing their recommendations until a consensus is reached. When revisions are invited, a fact-based synthesis statement explaining their decision and outlining what is needed to prepare a revision will be listed below. The following reviewer(s) agreed to reveal their identity: Christian Wallraven.

This manuscript takes a lot of inspiration from previous work and hence the question of novelty comes into play. The authors could do a little more to expand on the feature validation, which constitutes the main, novel aspect of the manuscript.

Overall, the manuscript reads well and discusses most of the relevant background literature well. Two aspects - mentioned above - that require attention is Experiment 2 in Gaissert et al., 2010, which actually includes a very similar setup [and motivation for designing it] in which rendered objects are rotated actively using a controller and viewed in 3D in an HMD. Second, the paper by Cooke et al., 2005a uses an identical setup for feature validation. These two studies should be discussed.

Concerning the analysis of the behavioral data, the “elbow” criterion that the authors cite is not actually used here, from the plot of the stress values in Fig. 3, the elbow would actually sit at a further distance, at either 3 or 4 dimensions. The authors should, therefore, to repeat their analysis with a dimensionality of 3.

This discussion also connects to the choice of stimuli: since the digital embryos do not have an underlying parameter space, identifying the dimensionality from the MDS reconstruction by resorting to the dimensionality of the producing physical parameter space is not possible. This should be discussed perhaps a bit more in detail since the authors highlight advantages of VP in the introduction, but do not discuss potential disadvantages of the VP creation process.

Next, and most importantly, I would like to discuss the validation of shape features. The authors extract 17 features from the 3D shapes and various 2D projections thereof. These are then used to create distance matrices, which are subjected to MDS, and then evaluated via Procrustes distance. Unfortuanately, it is not entirely clear why these specific features were selected - why these 17? Why not features that are closer to visual perception [such as 2D filters, higher-level features of deep neural networks,...] or haptic perception [Grasp-It features, etc]? How can the perceptual system(s) extract such features reliably [size, weight, curvature, intertial tensor??]? I think that the authors need to answer and discuss such questions [again, see Cooke et al., 2005a in which an identical setup was made for other types of features]

Concerning the processing of features, it is also not entirely clear for some how they were treated when applied to a mesh [for example, for curvature, was it averaged across the mesh?] Similarly, do the meshes have the same number of vertices? Wouldn't a re-tringulation [or “sanding”] result in very different values for some of the measures? How does number of vertices relate to “texture”?

Similarly, about finding the “best” number of features, it would be good to find some sort of confidence intervals for d (or to add also raw correlations of the similarity matrices). This could be done by Procrustes fitting of each feature space to each of the 25 individual spaces and then obtaining an estimate of the fit differences with the resulting 25 d-values.

If the extracted features are “valid” shape features, it would be great to show that they could be applied also in other situations. For example, could they be used to model the data in Gaissert at al?
