# Supplementary figures and images for: Visual and Tactile Sensory Systems Share Common Features in Object Recognition
Source: eNeuro. 2021 Oct 4;8(5):ENEURO.0101-21.2021. doi: 10.1523/ENEURO.0101-21.2021 (PMC8493885; doi:10.1523/ENEURO.0101-21.2021)

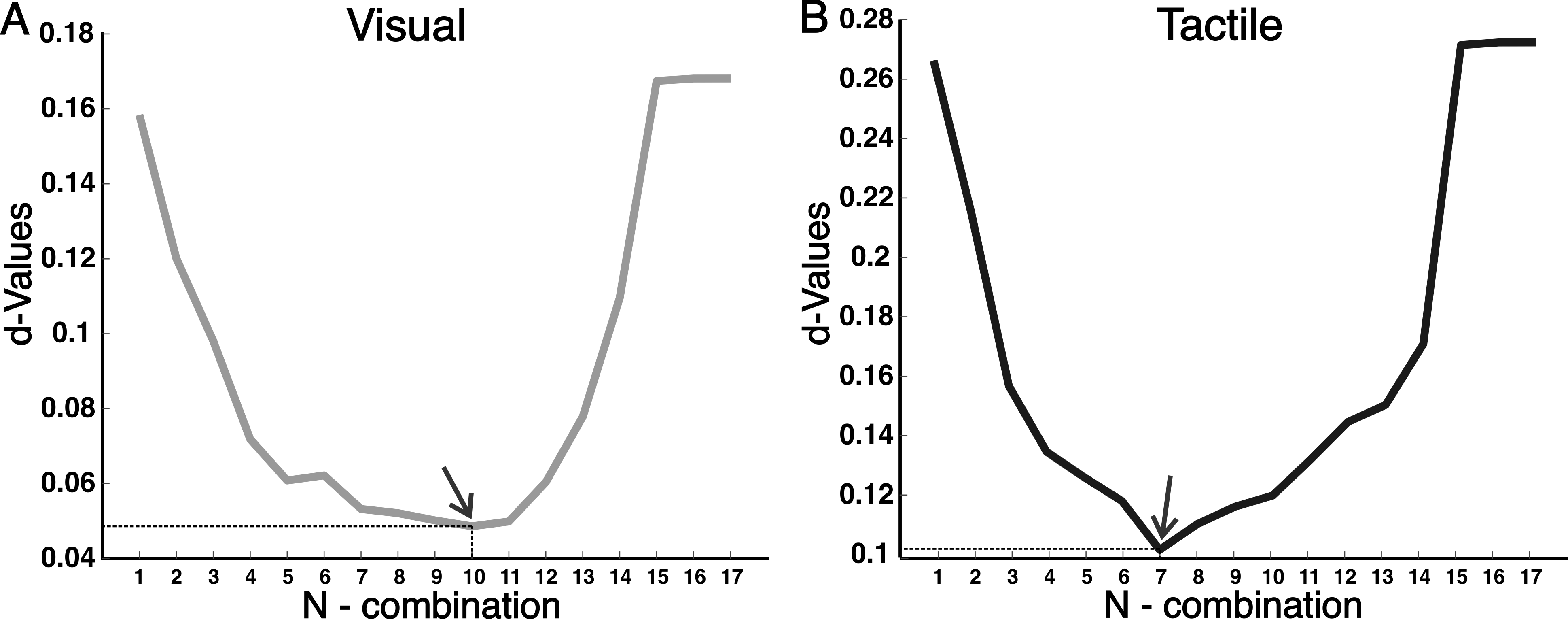

Supplement: Table 2-1 — The d values in Table 2 show that visual and tactile d values lead to U-shaped curves. A single feature or a combination of a few features led to high d values, and when the number of involving features rose, the d values again increased. It supports the notion that humans do not necessarily need to use all given features to reconstruct the perceptual spaces. Download Table 2-1, TIF file. [file enu-eN-NWR-0101-21-s03.tif]

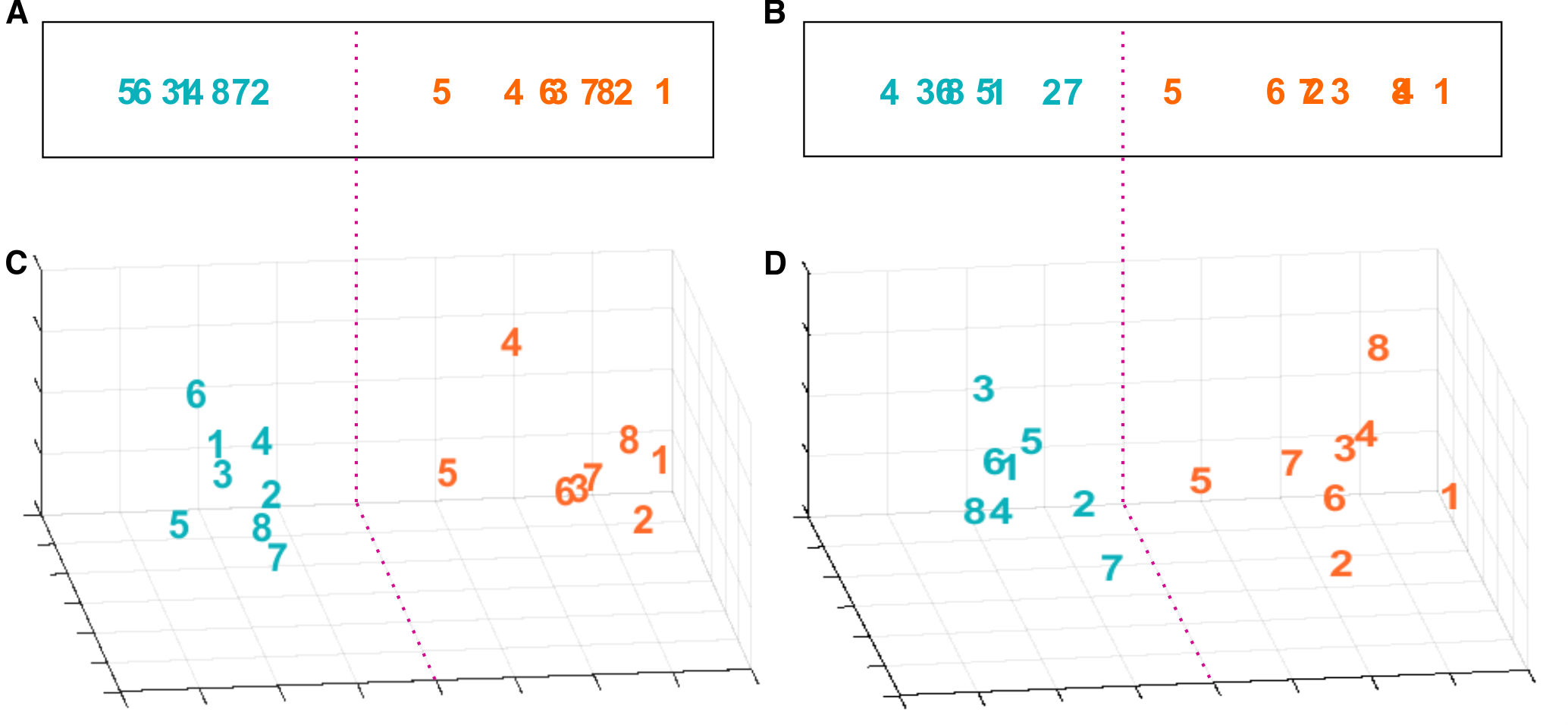

Supplement: Figure 3-1 — One- and three-dimensional visual and tactile perceptual spaces. A, One-dimensional visual perceptual space. B, One-dimensional tactile perceptual space. C, Three-dimensional visual perceptual space. D, Three-dimensional tactile perceptual space. The numbers refer to the object. Color codes two different categories. Download Figure 3-1, TIF file. [file enu-eN-NWR-0101-21-s02.tif]
